# Contribution of the *Staphylococcus aureus* Atl AM and GL Murein Hydrolase Activities in Cell Division, Autolysis, and Biofilm Formation

**DOI:** 10.1371/journal.pone.0042244

**Published:** 2012-07-31

**Authors:** Jeffrey L. Bose, McKenzie K. Lehman, Paul D. Fey, Kenneth W. Bayles

**Affiliations:** Department of Pathology and Microbiology, University of Nebraska Medical Center, Omaha, Nebraska, United States of America; University of Texas-Huston Medical School, United States of America

## Abstract

The most prominent murein hydrolase of *Staphylococcus aureus*, AtlA, is a bifunctional enzyme that undergoes proteolytic cleavage to yield two catalytically active proteins, an amidase (AM) and a glucosaminidase (GL). Although the bifunctional nature of AtlA has long been recognized, most studies have focused on the combined functions of this protein in cell wall metabolism and biofilm development. In this study, we generated mutant derivatives of the clinical *S. aureus* isolate, UAMS-1, in which one or both of the AM and GL domains of AtlA have been deleted. Examination of these strains revealed that each mutant exhibited growth rates comparable to the parental strain, but showed clumping phenotypes and lysis profiles that were distinct from the parental strain and each other, suggesting distinct roles in cell wall metabolism. Given the known function of autolysis in the release of genomic DNA for use as a biofilm matrix molecule, we also tested the mutants in biofilm assays and found both AM and GL necessary for biofilm development. Furthermore, the use of enzymatically inactive point mutations revealed that both AM and GL must be catalytically active for *S*. *aureus* to form a biofilm. The results of this study provide insight into the relative contributions of AM and GL in *S*. *aureus* and demonstrate the contribution of Atl-mediated lysis in biofilm development.

## Introduction

Murein hydrolases are hydrolytic enzymes that are involved in degradation, turnover, and maturation of bacterial peptidoglycan. As such, they play an essential role in cell division by assuring proper daughter cell separation. Due to their activity, they are tightly regulated to prevent accidental cell lysis, a phenomenon that has led them to be termed autolysins. In *S*. *aureus*, the most prominent murein hydrolase is AtlA, which is expressed as a 1,257 amino acid (137.5 kDa) pro-protein that is proteolytically cleaved into a 3.1 kDa signal sequence, a 17.6 kDa propeptide of unknown function, and two catalytically active proteins referred to as AM and GL [1]. AM is a 63.3 kDa *N*-acetylmuramyl-L-alanine amidase that cleaves the amide bond between the *N*-acetyl muramic acid in the murein backbone and L-alanine in the stem peptide [2]. Additionally, AM contains an enzymatic domain and two repeat domains that are involved in localization and substrate recognition [2], [3]. GL is a 53.6 kDa endo-β-*N*-acetylglucosaminidase that hydrolyzes the bond between *N*-acetyl-β-D-glucosamine and *N*-acetyl muramic acid and contains an enzymatic domain and a single repeat domain [1].

In addition to its role in cell division, several studies have identified the importance of the *atl* gene products (AtlA and AtlE in *S. aureus* and *S*. *epidermidis*, respectively) in the initial stages of biofilm formation [2], [4], [5]. Based on a combination of *in vivo* assays of biofilm formation, and *in vitro* interaction studies using purified protein, it has been hypothesized that these proteins function as adhesins involved in promoting the initial interactions of the bacteria to a variety of substrates [4], [6]. However, studies demonstrating the importance of cell lysis and extracellular DNA as a biofilm matrix molecule suggest an additional role for AtlA in biofilm formation. These studies have shown that *S*. *aureus* biofilms are disrupted by addition of DNase and that a lysis-deficient strain is attenuated in biofilm adherence [7]. In support of a role for AtlA-mediated lysis in biofilm development is the demonstration by Houston et al. that AM must be enzymatically active to contribute to biofilm adherence [5].

In the current study, we sought to examine in detail the relative contributions of AM and GL in autolysis and biofilm formation. Thus, we generated and characterized isogenic mutants specifically lacking AM and GL as a result of in-frame deletions, as well as mutants containing enzymatically inactive point mutations in both of these murein hydrolases. Using these mutants, we demonstrate the relative contributions of AM and GL in cell growth, whole-cell autolysis, and biofilm formation.

## Materials and Methods

### Strains and Media

*Escherichia coli* DH5α or DH5αλ*pir* were utilized for cloning, with the *pir*-containing strain used for plasmids bearing the R6Kγ origin of replication. *E*. *coli* was grown in LB medium with ampicillin (100 µg ml^−1^), spectinomycin (50 µg ml^−1^), and kanamcyin (100 µg ml^−1^) as needed for selection. *S*. *aureus* strains used in this study are listed in Supplementary Table S1 and were grown in TSB unless otherwise noted. When necessary for *S*. *aureus*, chloramphenicol (10 µg ml^−1^), tetracycline (5 µg ml^−1^), or erythromycin (5 µg ml^−1^) was added. Overnight cultures for experiments contained 1 µg ml^−1^ erythromycin for plasmid maintenance. Reagents were purchased from Sigma Aldrich (St. Louis, MO), EMD Chemical Inc (Gibbstown, NJ), or Becton, Dickinson, and Company (Sparks, MD).

### Molecular Genetic Techniques

Plasmids were purified using the Wizard Plus SV Minipreps DNA purification system (Promega Corporation, Madison, WI). Oligonucleotides (Supplementary Table S2) were synthesized by Integrated DNA Technologies (Coralville, IA) or the Eppley Molecular Biology Core Lab (University of Nebraska Medical Center, Omaha, NE). Klenow fragment, DNA ligase, and restriction enzymes were obtained from New England Biolabs (Beverly, MA). DNA fragments were recovered using the DNA Clean and Concentrator-5 Kit (Zymo Research, Orange, CA). PCR was performed with an Applied Biosystems GeneAmp PCR System 9700 (Life Technologies Corporation, Carlsbad, CA) using KOD DNA polymerase (Novagen, Madison, WI). PCR products were sequenced at the University of Nebraska Medical Center, to ensure that there were no unintended changes. Sequences were analyzed using Vector NTI (Invitrogen, Carlsbad, CA).

Chromosomal changes in *S*. *aureus* were engineered by allelic exchange. In-frame deletion plasmids were constructed by cloning upstream and downstream of the region to be removed into a temperature-sensitive *E*. *coli*-*S*. *aureus* shuttle vector. For example, ∼1000 bp of DNA upstream and 900 bp downstream of *atl* were amplified by PCR from the UAMS-1 chromosome. Primers were designed to add a BamHI recognition site on the 3′ and 5′ ends of the upstream and downstream products, respectively. These products were cloned into pCL52.2 such that the resulting plasmid contained the start and stop codons of *atl*, separated by the six base pair BamHI recognition sequence. Plasmids were generated in *E*. *coli* and transferred to the highly-transformable, restriction-deficient *S*. *aureus* strain RN4220. Transduction into clinical strains was performed using φ11 propagated on plasmid-containing RN4220. Transductants were maintained at the replication-permissive temperature of 30°C for plasmid maintenance and confirmation. To initiate recombination, freezer stocks of UAMS-1 containing the mutagenesis plasmid were struck onto TSA agar plates with the appropriate antibiotics and incubated at 45°C overnight. Large colonies were considered to be those that had undergone a single recombination event and were struck onto TSA for isolated colonies a second time, then incubated at 45°C overnight with selection. These single recombinants were used to inoculate 3 ml of TSB and incubated at 30°C without selection, to encourage a second round of recombination and plasmid loss. Following several days of serial dilutions, cultures were plated on TSA and the resulting colonies were replica patched onto TSA alone and TSA with antibiotic to identify those cells that had undergone a second recombination event and lost the plasmid. Antibiotic-susceptible colonies were screened by PCR to verify the presence of the mutant allele.

Due to the toxic nature of *atl* in *E*. *coli*, a two-plasmid system was employed to generate *atl*, *atl_AM_* and *atl_GL_* complementation plasmids. The first plasmid, pJB103, was generated by digesting pCN51 with XhoI and AvrII to remove the erythromycin resistance cassette (*ermC*), treating with Klenow to generate blunt ends, and self-ligating the remainder of the plasmid. Importantly, this plasmid contains a ColE1 origin of replication and ampicillin resistance gene for *E*. *coli*, as well as a pT181 origin of replication for *S*. *aureus*. The second plasmid, designated pJB104, was constructed by treating the *ermC* removed from pCN51 with Klenow, and ligating into pJB44 that had been Klenow-treated following digestion with NdeI and BglII. This plasmid encodes an R6Kγ origin of replication and spectinomycin resistance in *E*. *coli* as well as erythromycin resistance in *S*. *aureus*. An approximately 2600 bp fragment spanning 284 bp upstream of *atl* and the amidase portion of *atl* was amplified by PCR using primers JBATL14 and JBATL16. The PCR product was digested with PstI to generate two fragments. The first piece containing the *atl* promoter, signal peptide, and propeptide was cloned into pJB104 digested with PstI and SmaI to generate pJB106. Likewise, the second fragment containing the amidase encoding DNA was cloned into pJB103, producing pJB107. A stop codon (TAA) was added to the 3′-end of the PCR since the native stop codon for Atl is encoded by the glucosaminidase-encoding portion of the gene. To generate complement plasmid pJB111, pJB106 and pJB107 were digested with PstI and then ligated together. The ligation product was electroporated into RN4220. In order for the cells to grow, they must have a plasmid that contains a *S*. *aureus* origin of replication (pJB106) and encode erythromycin resistance (pJB107); therefore, due to plasmid design, colonies that grow on TSA supplemented with erythromycin must contain the fusion plasmid. The orientation of the fusion plasmid was determined by PCR and sequencing. A similar cloning scheme was used to generate an *atl* complement plasmid using UAMS-1 as a template to generate pJB141 and a glucosaminidase expressing plasmid, pJB135, using KB5002 as a template. See Supplementary Table S2 for construction details.

To generate alleles with point mutations, we began with cloned portions of *atl* in pCR-Blunt. Next, primers were designed to amplify around the entire plasmid, with the point mutations incorporated into one of the primers. Since T4 DNA ligase requires a 5′ phosphate, these primers were 5′-phosphorylated so the resulting PCR products could be self-ligated, circularizing the PCR product, and generating a new plasmid with the desired change. Ligation products were treated with DpnI to remove the original template prior to transformation. This mutant allele was then moved into pJB103 followed by fusion to pJB106. For example, the AM^H263A^ mutation was produced by amplifying around pJB110, using primer JBATL31 incorporating a GCA codon instead of the wild-type CAT codon to generate pJB113. The partial *atl* gene containing the AM^H263A^ allele was moved from pJB120 into pJB103, which when fused to pJB106 would generate an AM^H263A^ complement plasmid.

### Growth Analysis

Overnight cultures were diluted to an OD_600_ of 0.1 in a 250-ml flask with 25 ml of TSB and incubated at 37°C with shaking (250 rpm). At intervals, samples were removed, diluted as necessary, and optical density at 600 nm was measured using an Ultrospec 4300 *pro* (GE Healthcare Biosciences, Pittsburg, PA) spectrophotometer. In mid-exponential phase, 3 hrs post-inoculation, samples were removed and visualized by light microscopy or confocal microscopy to estimate cell cluster size. In addition, samples were examined using flow cytometry at the UNMC Cell Analysis Facility using a BD FACSCalibur with forward scatter and a 488 nm laser as a representation of cluster size. Flow cytometry data were analyzed using CellquestPro software.

### Static Biofilm Assays

Static biofilm assays were performed using a variation of a method previously described [8]. A single colony was used to inoculate 2 ml of static biofilm medium (TSB with 0.5% glucose) and incubated overnight at 37°C with shaking (250 rpm). Cultures were diluted to an OD_600_ of 0.05 and 200 µl was added to a Costar 3596 96-well polystyrene cell culture plate (Corning Inc., Lowell, MA) that had been pretreated with 200 µl of human plasma (Sigma Aldrich, cat# P9523–5 ML reconstituted in 20 ml of carbonate-bicarbonate buffer) overnight at 4°C. Human plasma is commonly used in *S*. *aureus* biofilm assays and is necessary for UAMS-1 biofilm formation in microtiter plates (unpublished observation and [8]). The plate was incubated statically at 37°C for 24 hrs. Following incubation, the wells were washed twice with 200 µl of PBS. For consistency, washes were performed using an electronic multichannel pipette. Washed samples were treated with 100 µl 100% ethanol for 2 min and stained with 100 µl 0.41% crystal violet in 12% ethanol for 2 min. Excess stain was removed by three washes of 200 µl PBS. The resulting biofilms were quantified with multiple readings per well at OD_595_ using a Victor^3^V (Perkin Elmer, Oak Brook, IL). Wells containing media alone were used as negative controls. Biofilm quantification was corrected by subtracting the average OD_595_ of the media only-containing wells that had been treated the same as the wells with biofilms. The amount of biofilm produced by each strain was calculated as a percentage of that produced by the wildtype strain and was determined based on the average readings from four wells.

eDNA was isolated using a previously described method [7] with modification. Briefly, cells were grown as described for static biofilm assays. At 24 hrs, the supernatants from six wells per strain were removed and the cells were resuspended in a high salt Tris buffer (50 mM Tris-HCl, 10 mM EDTA, and 500 mM NaCl, pH 8.0). Following centrifugation, 100 µl of supernatant were transferred to a fresh tube containing 300 µl of TE buffer (10 mM Tris-HCl, 1 mM EDTA, pH 8.0) and then extracted with equal volumes of phenol/chloroform/isoamyl alcohol (25∶24:1) and chloroform/isoamyl alcohol (24∶1). Samples were then mixed with 3 volumes of cold 100% ethanol and 1/10 volume 3M Na-acetate and incubated overnight at −20°C. The following day, samples were centrifuged at 4°C, washed with cold 75%, air dried, and resuspended in 20 µl TE. eDNA was quantified using real-time PCR with *gyrA* primers as previously described [7]. eDNA per OD was calculated and is reported as a percentage of that produced by the wild-type strain.

### Whole-cell Autolysis Assays

Autolysis assays were performed using a modification of previously published methods [9]. Overnight cultures were diluted to an OD_600_ of 0.1 in 12.5 ml TSB +1 M NaCl in a 125-ml flask. Cultures were incubated at 37°C with shaking at 250 rpm. After 3 hrs, the cultures were centrifuged for 10 min at 4100 rpm, washed twice with 12.5 ml 4°C water, and resuspended in autolysis buffer (50 mM Tris-HCl (pH 7.2) with 0.05% Triton X-100) to an OD_600_ less than 1.0. Subsequently, 1 ml of cell suspension was placed in a cuvette and covered in parafilm. After obtaining an initial OD_580_ reading, the cuvettes were incubated at 30°C with shaking at 250 rpm. At 30 min intervals, samples were mixed by inversion and the OD_580_ was measured using an Ultrospec 4300 *pro* (GE Healthcare Biosciences) spectrophotometer. Data are reported as the percent of the initial OD_580_ for each sample.

### Murein Hydrolase Assays

Overnight cultures grown in TSB were diluted to an OD_600_ of 0.1 in 10 ml of TSB in a 125-ml flask. Cultures were incubated for three hours at 37°C with shaking at 250 rpm to isolate proteins from mid-exponential phase. Ten ml of culture was centrifuged and the supernatants were concentrated using Amicon Ultra-4 3,000 MWCO filters (Millipore Corporation) to approximately 250 µl and frozen at −80°C until used. Protein concentrations were determined using the Bio-Rad Protein Assay (Bio-Rad Laboratories, Inc.) following the manufacturer’s instructions. Next, the murein hydrolase profiles of these concentrated extracellular proteins were analyzed by zymographic analyses essentially as previously described [10], [11]. Three micrograms of total protein for each sample set was separated using SDS-PAGE on a 12% acrylamide gel containing 1 mg ml^−1^ *Micrococcus lysodeikticus* ATCC No 4698 cells (Sigma Aldrich, St. Louis, MO) or 2.5×10^9^ CFU ml^−1^ of heat-killed mid-exponential phase *S*. *aureus* UAMS-1 at 100 V for approximately 2 hrs with a Mini-PROTEAN Tetra Electrophoresis System (Bio-Rad). Following electrophoresis, the gel was washed with reaction buffer [25 mM Tris-HCl (pH 8.0) with 1% Triton X-100] for 30 min and then incubated in reaction buffer overnight, statically at 37°C. Gels were rinsed three times with water and stained with 1% methylene blue in 0.01% KOH. Quantitative murein hydrolase assays were performed essentially as described previously [10], [12] except that cells were grown under the same conditions as the autolysis assays. Culture supernatants were collected and concentrated using Amicon Ultra-4 3,000 MCO filters (Millipore Corporation, Billerica, MA). The concentrated proteins were quantified using the Bio-Rad Protein Assay (Bio-Rad Laboratories, Inc., Hecules CA). Assays were performed in 1 ml of 1.0 mM Tris (pH 8.0) containing 100 µg of proteins and 0.2 mg ml^−1^ *M. lysodeikticus* cells. The cells were incubated at 37°C at 250 rpm and OD_580_ readings were taken every 30 minutes. Data are reported as the percent of the initial OD_580_ for each sample.

### Western Blot Analysis

Proteins concentrated and quantified for zymogram analysis were also subjected to immunoblot analysis. Again, 3 µg of total protein for each sample was separated using SDS-PAGE on a 12% acrylamide gel at 100 V for 1 hour and 40 minutes. The proteins were then transferred onto a 0.1 micrometer pore Whatman Protran nitrocellulose transfer membrane overnight at 20 V at 4°C. After transfer, the nitrocellulose membrane was washed 3 times with TBS (150 mM NaCl, 10 mM Tris-HCl) and blocked for 1 hour with 1% casein blocking solution (150 mM NaCl, 10 mM Tris-HCl, 0.1% Tween, 1% casein). To block any non-specific antibody binding by protein A, a second blocking solution, with 1∶1000 dilution of unrelated IgG antibodies, was added for 1 hour. The blot was washed twice with TBSTT (500 mM NaCl, 20 mM Tris-HCl, 0.05% Tween-20, 0.2% Triton-X 100) and once with TBS. Subsequently, the blot was incubated for 1 hour at room temperature with a 1∶1000 dilution (in 1% casein blocking solution) of antibodies specific to the catalytic domain of amidase or glucosaminidase. Finally, the blot was washed twice with TBSTT and once with TBS, followed by a 1-hour room temperature incubation with anti-rabbit IgG secondary antibodies conjugated to alkaline phosphatase (Sigma Aldrich). The blot was washed 5 times with TBSTT and then developed via a colormetric reaction, using 1-Step NBT/BCIP solution (Thermo Scientific).

### Statistical Analysis

Biofilm data were analyzed by performing a one-way ANOVA with a Tukey post-test using GraphPad Prism version 5.0 for Windows, www.graphpad.com (GraphPad Software, La Jolla, CA).

## Results

### Construction of Atl Deletion Mutants

**Figure 1:**
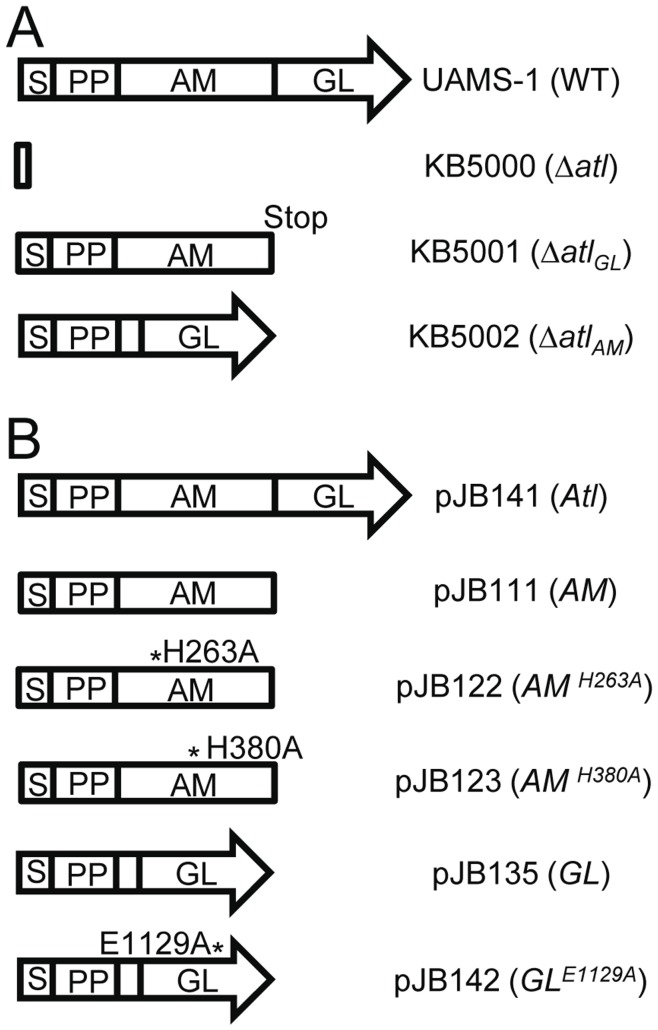
Summary of chromosomal and plasmid-encoded *atl* alleles. A) Representation of chromosomally-encoded alleles. Wildtype *atl* (UAMS-1) encodes a signal peptide (S), propeptide (PP) of unknown function, an amidase (AM) domain, and a glucosaminidase (GL) domain. In-frame deletions were made by allelic exchange. “Stop” indicates that a translational stop sequence (TAA) was added during the deletion of GL. Genotype for each strain is show in parentheses. B) Depiction of plasmid-expressed constructs with protein made indicated in parentheses. All The symbol “*” identifies the location of point mutations generated in AM or GL with numbers based upon the amino acid residue position in full-length Atl. Plasmid-encoded alleles are under control of the native promoter.

The *S. aureus* AtlA murein hydrolase is a bifunctional protein that is proteolytically cleaved into two active enzymes, an amidase (AM) and a glucosaminidase (GL). While previous studies have given insight into the function of AtlA in cell wall metabolism and biofilm formation [1], [2], [3], [4], [5], [13], [14], [15], no studies have examined the individual contributions of AM and GL in these processes. Specifically, these previous studies typically use complete *atl* knockouts and complementation via plasmid-encoded *S*. *epidermidis atl* or AM alone, with the activity of GL largely overlooked. Therefore, we constructed individual in-frame deletions of AM and GL in *S*. *aureus* UAMS-1, providing isogenic mutants of a well-characterized clinical isolate (see Fig. 1 and Supplemental Table S1). As described in the Materials and Methods, the entire *atl* ORF was replaced by a BamHI endonuclease recognition site yielding KB5000. Likewise, in-frame-deletions of the GL- and AM-encoding regions of *atl* were created, resulting in KB5001 and KB5002, respectively. For the latter, the N- and C-terminal fifteen amino acids of AM were retained to leave any protease recognition sites intact, allowing proper processing of the downstream GL, but removing the previously described catalytic domain and repeat domains of AM.

To confirm that any observed phenotypes were due to the generated mutations, we also created complement plasmids. Similar to previous groups [1], [4], we had difficulties cloning intact, full-length *atl* in *E*. *coli,* likely due to a detrimental effect of overexpression of AtlA. To overcome this, we generated a two-plasmid system in *E*. *coli* (described in Materials and Methods) that disconnected the *atl* promoter and 5′-end of *atl* from the enzyme-encoding regions. Once combined in *S*. *aureus*, the fusion plasmid yielded a functional complement plasmid with *atl* under the control of its native promoter. Using this method, we successfully generated *atl*, *atl_AM_*, and *atl_GL_* complement plasmids with expression of full-length AtlA, AM, and GL, respectively, under the control of the native *atl* promoter.

UAMS-1 is a low-passage methicillin-susceptible USA200 strain isolated from an osteomyelitis infection. However, strains of *S*. *aureus* behave quite differently and it is often prudent to examine observed phenotypes in multiple genetic backgrounds. While the importance of AtlA has been documented in several strains, we sought to confirm the function of AtlA in a second clinically relevant background. Therefore we chose to generate an *atl* deletion strain in a derivative of the community-associated methicillin resistant USA300 isolate LAC. Since USA300 LAC contains a 27 kb plasmid (LAC-p03) encoding erythromycin resistance [16], we cured this plasmid by screening for the spontaneous loss of erythromycin resistance and designated this strain LAC-13C. Due to sequence divergences in DNA flanking *atl*, separate allelic exchange plasmids were constructed to generate the LAC-13C Δ*atl* mutant KB4051 (Supplementary Table S2).

### The Effect of *Atl* Mutations on Cell Growth

**Figure 2:**
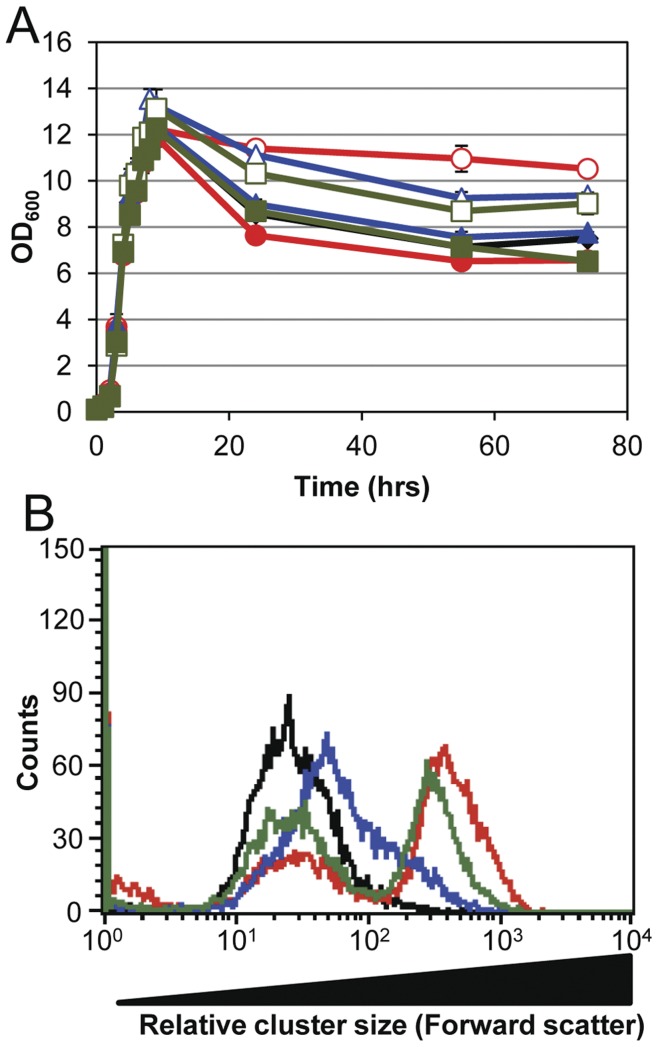
Growth characteristics of the *atl* mutants. A) UAMS-1 (black solid diamonds), Δ*atl* mutant KB5000 (red empty circles), KB5000 with pJB141 (red solid circles), Δ*atl_AM_* mutant KB5002 (green empty square), KB5002 with pJB111 (green solid square), Δ*atl_GL_* mutant KB5001 (blue empty triangle), and KB5001 with pJB135 (blue solid triangle) were grown in TSB with a 1∶10 media to volume ratio at 37°C with shaking (250 rpm). Data represent the mean (n = 2) with standard error. B) Relative cluster size as determined by flow cytometry of mid-exponential phase UAMS-1 (black), Δ*atl* mutant KB5000 (red), Δ*atl_AM_* mutant KB5002 (green), and Δ*atl_GL_* mutant KB5001 (blue). UAMS-1 and mutants contain the control vector, pJB128.

Despite being a prominent murein hydrolase, *atl* mutants in *S*. *epidermidis* [13] and *S*. *aureus* [2] show little differences in growth rate when measured by optical density, presumably as a result of the expression and activity of other murein hydrolases. However, *atl* mutants demonstrate a clustering phenotype due to incomplete daughter cell separation [2], [17]. To examine the functions of both catalytic domains of AtlA, we compared the growth characteristics of the Δ*atl* (KB5000), Δ*atl_AM_* (KB5002), and Δ*atl_GL_* (KB5001) in-frame deletion mutants. The Δ*atlA* mutant showed no difference in absorbance-based measure of growth during exponential phase (mean  = 32.9 min gen^−1^, std error  = 1.0) (Fig. 2A) compared to WT (31.2 min gen^−1^, std error  = 0.5). Similar to the Δ*atl* mutant, the Δ*atl_AM_* (mean  = 33.5 min gen^−1^, std error  = 0.4) and Δ*atl_GL_* (mean  = 31.4 min gen^−1^, std error  = 0.1) mutants exhibited comparable maximal growth rates (Fig. 2A). Similar growth rates were also observed in a Δ*atl* mutant of a second clinical isolate, LAC, compared to its parental strain (Fig. S2A). Because *S*. *aureus* undergoes stationary-phase lysis, we also examined these mutants during this phase of the growth cycle. As shown in Figure 2A, the Δ*atlA* mutant exhibited a nearly complete loss of stationary-phase lysis. Both the Δ*atl_AM_* and Δ*atl_GL_* mutants had an intermediate lysis phenotype when compared to the wild-type and Δ*atl* strains, indicating that there was an additive effect of AM and GL on cell lysis. As shown in Figure 2A, the decreased lysis seen in the mutant strains could be restored to wild-type levels with the presence of the corresponding complementation plasmids.

Since mutations in *atl* are known to cause a defect in daughter cell separation, the mutant strains were also examined in mid-exponential phase by confocal microscopy (Fig. S1) to evaluate cluster size differences. While the wild-type strain consisted mostly of clusters containing 4 cells, with some as high as 8 cells, the Δ*atl* mutant was found to form two distinct groups containing different cluster sizes. The first group contained smaller cell clusters with an average of 3 cells per cluster but the majority of cells were found in clusters averaging 32 cells (data not shown). This clustering phenotype could also be detected using flow cytometry (Fig. 2B), which clearly indicated two cluster sizes produced by the Δ*atl* mutant. The Δ*atl_AM_* and Δ*atl_GL_* were also examined to characterize the individual contributions of AM and GL in daughter cell separation. These mutants had phenotypes that were distinguishable from both the wild-type and Δ*atl* mutant. The Δ*atl_AM_* mutant was comparable to the Δ*atl* strain, with two groups of clusters as determined microscopically and using flow cytometry (Fig. 2B). Specifically, the first group contained clusters of approximately 3 cells and a second, larger group consisting of clusters averaging 23 cells. Interestingly, the Δ*atl_AM_* mutant had more of the smaller clusters than the Δ*atl* mutant, presumably due to the presence of GL. The Δ*atl_GL_* strain was more similar to UAMS-1, with only one cluster group (Fig. 2B) containing 8 cells on average. All of these cluster phenotypes could be restored to wild-type when AtlA, AM, and GL were expressed from plasmids in the corresponding mutant strains (Fig. S3). These data are the first to clearly demonstrate that daughter cell separation in *S. aureus* is a result of the additive contributions of AM and GL.

### Contribution of AM and GL to Murein Hydrolase Activity

**Figure 3:**
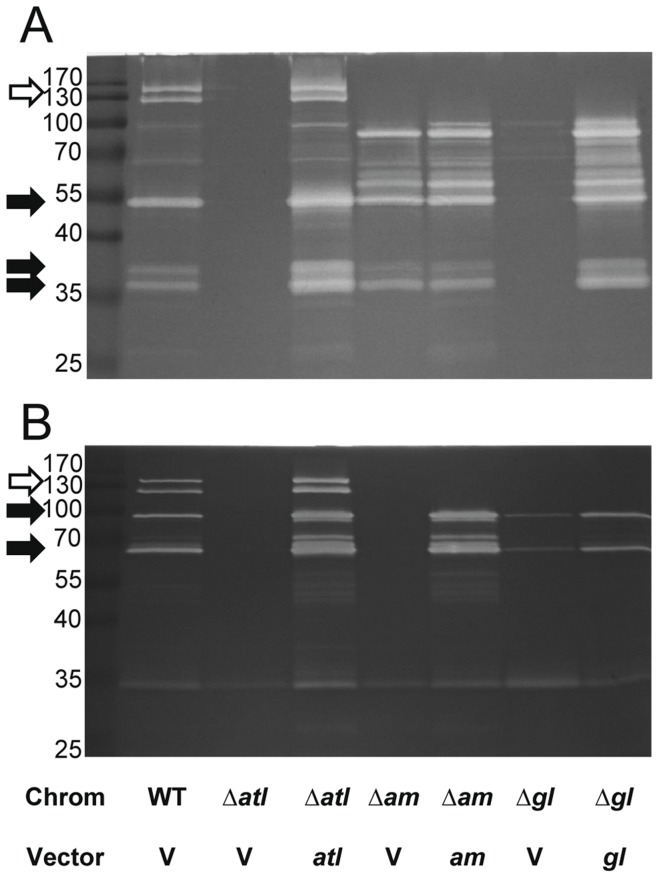
Zymography UAMS-1 *atl* mutants and complement plasmids. Three µg of total extracellular proteins were separated by SDS-PAGE on 12% acrylamide gels containing either A) *Micrococcus* or B) *S*. *aureus* as a substrate. “Chrom” denotes chromosomal genotype for UAMS-1 (WT), Δ*atl* mutant KB5000 (Δ*atl*), Δ*atl_AM_* mutant KB5002 (Δ*am*), or Δ*atl_GL_* mutant KB5001 (Δ*gl*). “Vector” indicates whether the strain carried vector control pJB128 (V), *atl_AM_* complement plasmid pJB111 (*am*), or *atl_GL_* complement plasmid pJB135(*gl*). Black arrows denote *GL* and *AM*-specific bands in WT for panels A and B, respectively. Empty arrow indicates the two bands in WT containing both AM and GL in both A) and B).

To further understand the individual contributions of AM and GL to cell lysis, the deletion mutants were examined for murein hydrolase activity using standard zymographic and whole cell lysis assays. AtlA is known to undergo several proteolytic cleavage events [1], [4], [11] and extracellular proteins isolated from the mid-exponential phase culture supernatants showed multiple murein hydrolase bands that were apparently AtlA-related, since they were absent in the Δ*atl* mutant (Fig. 3). Indeed, only a single non-AtlA murein hydrolase of approximately 35 kDa could be seen in the Δ*atl* mutant (Fig. 3B). Since the use of *Micrococcus* or *Staphylococcus* cells can be used to differentiate between AM and GL activity [1], [18], we examined the *atl* mutants by zymography using each substrate to correlate Atl-dependent bands to their respective enzyme AM or GL. The use of the mutants in these assays also allowed us to identify each band as AM and/or GL. Using *Micrococcus* cells as a substrate (Fig. 3A), revealed that three of the AtlA-related bands are GL-specific. The molecular mass of the first is slightly less than 55 kDa, which corresponds to GL alone. Two other bands, slightly larger than 35 kDa were also GL specific and correlate with the approximate size (36 kDa) of the catalytic domain of GL but missing the repeat domain. In contrast, the use of *S. aureus* as a substrate (Fig. 3B), revealed two bands that were AM-specific. The larger band (slightly less than 100 kDa), likely corresponds to AM with the propeptide still attached (81 kDa), while the lower molecular weight band (slightly less than 70 kDa), corresponds to mature amidase (63 kDa). As expected, there were two bands of high molecular weight (134 kDa and 117 kDa) that were detected using both substrates, indicating the presence of both AM and GL activity. The 117 kDa fragment most likely represented the AM-GL peptide, whereas the 134 kDa fragment represented the AM-GL peptide containing the propeptide. Furthermore, analysis of the Δ*atl_AM_* and Δ*atl_GL_* mutants and the use of different murein hydrolase substrates allowed us to confirm which bands were processed products of both AM and GL. In addition, mutants harboring the complementation plasmids were analyzed and found to restore the production of the expected murein hydrolases but at levels greater than the chromosomal copies of UAMS-1. These assays allowed us to better characterize the processed derivatives of AtlA and demonstrate that the mutants generated are expressing the products we expected.

**Figure 4:**
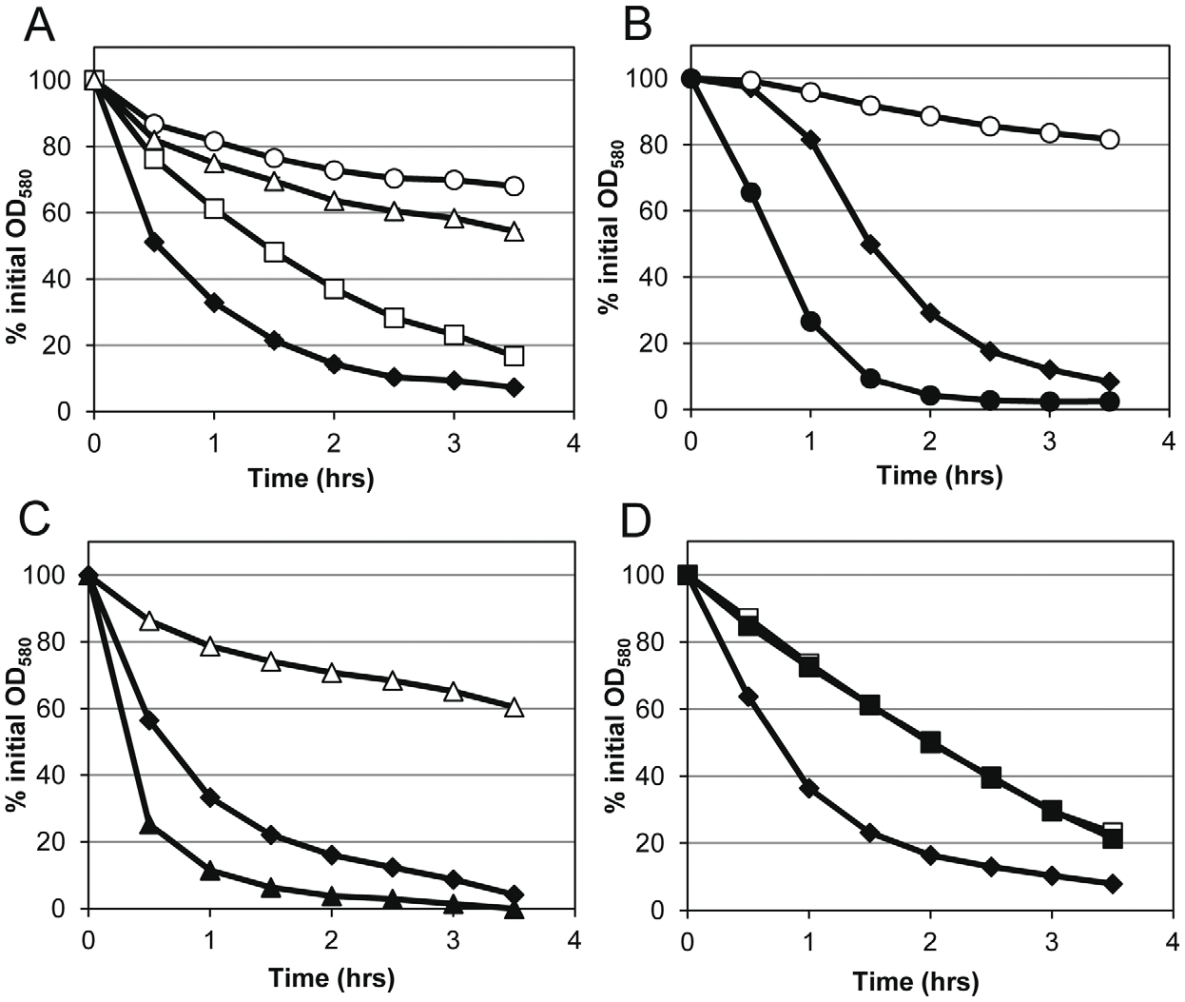
Autolysis of *atl* mutants. Triton X-100 induced autolysis of mid-exponential phase cultures grown in TSB with 1 M glucose at a 1∶10 media to flask volume ratio. A) comparison of UAMS-1 (solid diamonds), Δ*atl* mutant (empty circles), Δ*atl_AM_* mutant (empty triangles), and Δ*atl_GL_* mutant (empty squares). B) UAMS-1 (solid diamonds), Δ*atl* mutant (empty circles), and mutant with *atl* complement plasmid pJB141 (solid circles). C) UAMS-1 (solid diamonds), Δ*atl_AM_* mutant (empty triangles), and mutant with *atl_AM_* complement plasmid pJB111 (solid triangles). D) UAMS-1 (solid diamonds), Δ*atl_GL_* mutant (empty squares), and mutant with *atl_GL_* complement plasmid pJB135 (solid squares). B–D) UAMS-1 and mutants contain vector alone plasmid pJB128. Data represent the mean (n = 3 for panel A, n = 2 for B–D) with standard error (bars are smaller than symbols).

To examine the activity of AM and GL in intact cells, the mutant strains were tested in triton X-100-induced whole-cell autolysis assays. Consistent with the overall level of murein hydrolase activity observed in the zymographic analyses, UAMS-1 underwent rapid lysis while the Δ*atl* mutant was lysis deficient (Fig. 4A), a phenotype that could be over-complemented by *atl* when expressed from the multicopy plasmid, pJB141 (Fig. 4B). Again, comparable effects of the Δ*atl* mutation in the *S. aureus* LAC strain on autolysis were also observed (Fig. S2B). Similar to the cluster size findings, the GL mutation caused only a moderate effect on autolysis, while the AM mutant behaved similar to the complete *atl* deletion mutant (Fig. 4A), indicating that *S*. *aureus* cells are more sensitive to AM than GL. Similar to the *atl* complementation plasmid, the expression of AM from the multicopy plasmid, pJB111, restored activity beyond the level of the wild-type cells (Fig. 4C). However, for reasons that are unclear, overexpression of GL from pJB135 did not complement the autolysis defect (Fig. 4D) of the Δ*atl_GL_* mutant despite the observation that GL overexpression could be visualized by zymography (Fig. 3).

### AM and GL are Necessary for Biofilm Formation

**Figure 5:**
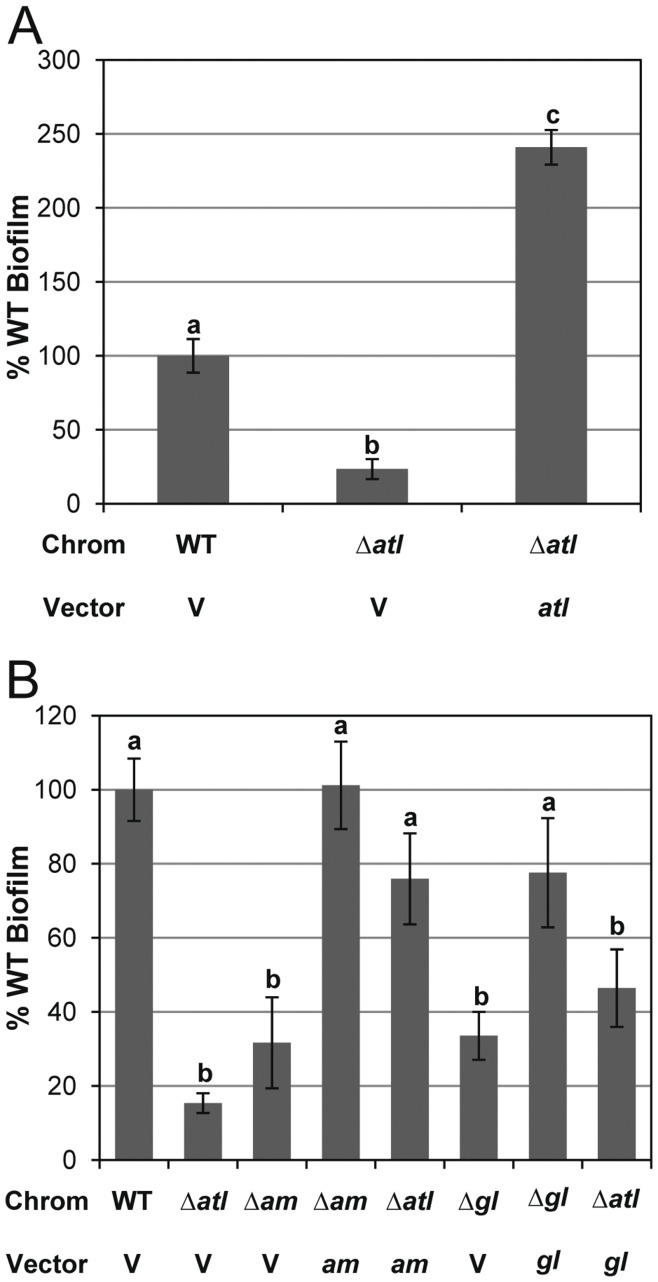
Static biofilm assays of *atl* mutants. Quantified crystal violet stained 24-hr adherent biofilms grown in TSB supplemented with 0.25% glucose. “Chrom” denotes chromosomal genotype for UAMS-1 (WT), KB5000 (Δ*atl*), Δ*atl_AM_* mutant KB5002 (Δ*am*), or Δ*atl_GL_* mutant KB5001 (Δ*gl*). “Vector” indicates whether the strain carried vector control pJB128 (V), *atl* complement plasmid pJB141 (*atl*), *atl_AM_* complement plasmid pJB111 (*am*), or *atl_GL_* complement plasmid pJB135(*gl*). Values were calculated using the average of the WT biofilms and represent the mean (n = 4) with standard error. For A) “a”, “b”, and “c” are significantly different from each other (p<0.01). For B) a and b denote not significantly or significantly different (p<0.05) than WT, respectively. See Results for more detailed statistical analysis.

It has been previously shown that Atl is necessary for biofilm formation in *S. aureus* and *S. epidermidis* [2], [4], [5], [13]. In addition, Heilmann et al. demonstrated that a plasmid expressing the *atlE*-encoded AM alone could complement biofilm formation in *S*. *epidermidis*. However, a detailed analysis of AM and GL in *S. aureus* biofilm has not been previously reported. Thus, we first took advantage of our mutant strains to assess the impact of these murein hydrolases on biofilm formation in static biofilm assays. As shown in Figure 5A, the Δ*atl* mutant (KB5000) produced adherent biomass at levels that were only 23% of that produced by the parental strain (p<0.01), a defect that was restored beyond wild-type levels (241% relative to wild type; p<0.001) when *atl* was expressed from a plasmid. Likewise, that Δ*atl* mutant of LAC-13C was also found to produce 24% of the biofilm produced by the parental strain (Fig. S2D). Interestingly, the AM and GL mutants formed significantly less biofilm (31.7% and 33.6% adherent biomass compared to wildtype, respectively; both p<0.01), demonstrating that both AM and GL are essential for optimal biofilm attachment (Fig. 5B). While not statistically significant, the Δ*atl_AM_* and Δ*atl_GL_* mutants trended towards a slightly elevated amount of biofilm formation when compared to the Δ*atl* strain. The biofilm adherence defect in the AM mutant could be completely restored when expressed from the plasmid, pJB111 (101% relative to wildtype), while the GL defect was complemented to 77.6% of wild-type levels. In addition, the expression of AM or GL from pJB111 and pJB135, respectively, in the Δ*atl* strain restored biofilm formation beyond the levels of the individual deletions (but not to wild-type levels), demonstrating that overexpression of either AM or GL can partially compensate for the loss of the other enzyme. Moreover, this confirms a previous study [4] where AM appeared sufficient for biofilm formation in an *atl* knockout and highlights the importance of studying the individual contributions of AM and GL in a low copy context, as their overexpression will mask the activity of the other enzyme. For example, the use of individual disruptions of AM and GL as described here, allowed us to clearly demonstrate a function for GL in biofilm formation.

eDNA has been shown to be an essential constituent of *S*. *aureus* biofilm formation [5], [7]. We predicted that the *atl* mutant cells found in the wells containing biofilm would have decreased eDNA due to decreased lysis. Because the Δ*atl*, Δ*atl_AM_*, and Δ*atl_GL_* mutant strains produce very little biofilm, we examined the cell-associated eDNA of cells grown under biofilm-forming conditions as has been described previously [7]. Under these conditions, the wild-type strain was found to contain 240 pg per OD eDNA (n = 6). However, the Δ*atl*, Δ*atl_AM_*, and Δ*atl_GL_* mutants only contained 16%, 12% and 16%, respectively, when compared to the wild-type strain demonstrating that the *atl* mutants have much reduced eDNA.

### Production of Enzymatically Inactive Variants of AM and GL

The contribution of AtlA to biofilm formation has been attributed to its activity as an adhesin by promoting binding of the bacteria to host proteins or surfaces in the initial stages of biofilm development. However, more recent evidence suggests that the function of AtlA in biofilm formation also involves its function in lysis of the cells and release of genomic DNA into the biofilm matrix during early biofilm formation. To test this, we generated point mutations in *atl* that rendered AM and GL enzymatically inactive and made use of our mutants and expression constructs to determine if these mutant enzymes could complement the AM and GL mutant biofilm defects. In this respect, Yokoi et al. [19] have identified amino acids that are critical for AM and GL activity. Based on these results, we generated both an AM^H263A^ and AM^H380A^ variant, which should render AM inactive, and expressed them in the plasmids pJB122 and pJB123, respectively. Importantly, it was recently shown that H263 is part of a Zn-binding pock et al ong with H380, while the AM^H263A^ variant of *S*. *epidermidis* AtlE can still bind substrates and is deficient only in substrate cleavage [20]. While no activity associated with the AM^H263A^ variant was detected by zymographic assays, the AM^H380A^ mutant (Fig. S4) produced a reduced but detectable level of murein hydrolase activity. In addition we produced a GL^E1129A^ and demonstrated its loss of activity by zymography (Fig. S4). As shown in Figure S4, the reduced activity of these AM and GL variants was not due to lack of expression, as they could be visualized by western blot analysis at levels comparable to those produced by the wild-type alleles.

**Figure 6:**
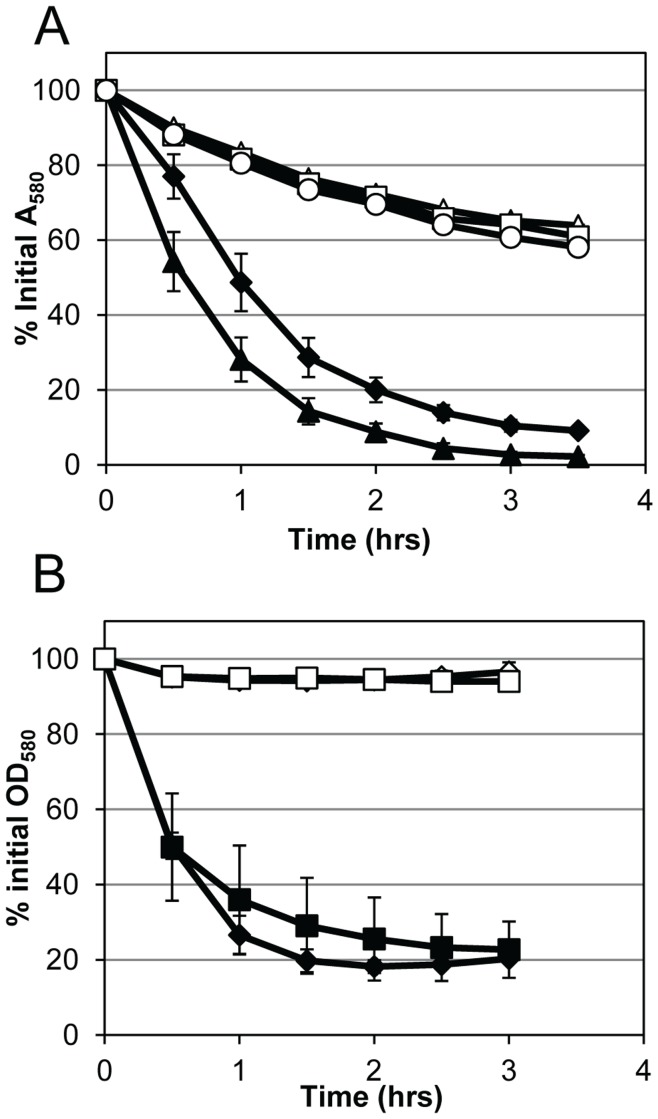
Effect of *atl* point mutations on murein hydrolase activity. A) Triton X-100 induced autolysis of mid-exponential phase cultures grown in TSB with 1 M NaCl at a 1∶10 media to flask volume ratio. UAMS-1 pJB128 (solid diamonds), Δ*atl_AM_* mutant pJB128 (empty triangles), mutant with *atl_AM_* complement plasmid pJB111 (solid triangles), mutant with *atl_AMH263A_* complement plasmid pJB122 (empty square), and mutant with *atl_AMH380A_* complement plasmid pJB123 (empty circles). B) Quantitative murein hydrolase activity of culture supernatants on *Micrococcus* cells. UAMS-1(pJB128) (solid diamonds), Δ*atl_GL_* mutant KB5001 (empty diamonds), mutant with *atl_GL_* complement plasmid pJB135 (solid squares), and mutant with *atl_GLE1129A_* complement plasmid pJB142 (empty squares). Data represent the mean (n = 2) with standard error.

We then sought to confirm the inactivity of the AM and GL point mutations in whole-cell autolysis assays. As seen in Figure 6A, both the AM^H263A^ and AM^H380A^ variants demonstrated dramatically reduced autolysis and resembled the AM deletion strain. Since wild-type GL expressed from a plasmid did not show activity in these assays, we examined the activity of GL and the GL^E1129A^ mutation on the GL-preferred substrate, *Micrococcus* cells, using supernatant proteins in quantitative murein hydrolase assays. While there was a higher level of variation in these assays, we saw that GL expressed from pJB135 was able to restore the murein hydrolase defect seen in the Δ*gl* mutant (Fig. 6B). As anticipated, the GL^E1129A^ variant was unable to complement the murein hydrolase activity and resembled the Δ*gl* mutant.

### Enzymatic Activity of AM and GL is Essential for Biofilm Formation

**Figure 7:**
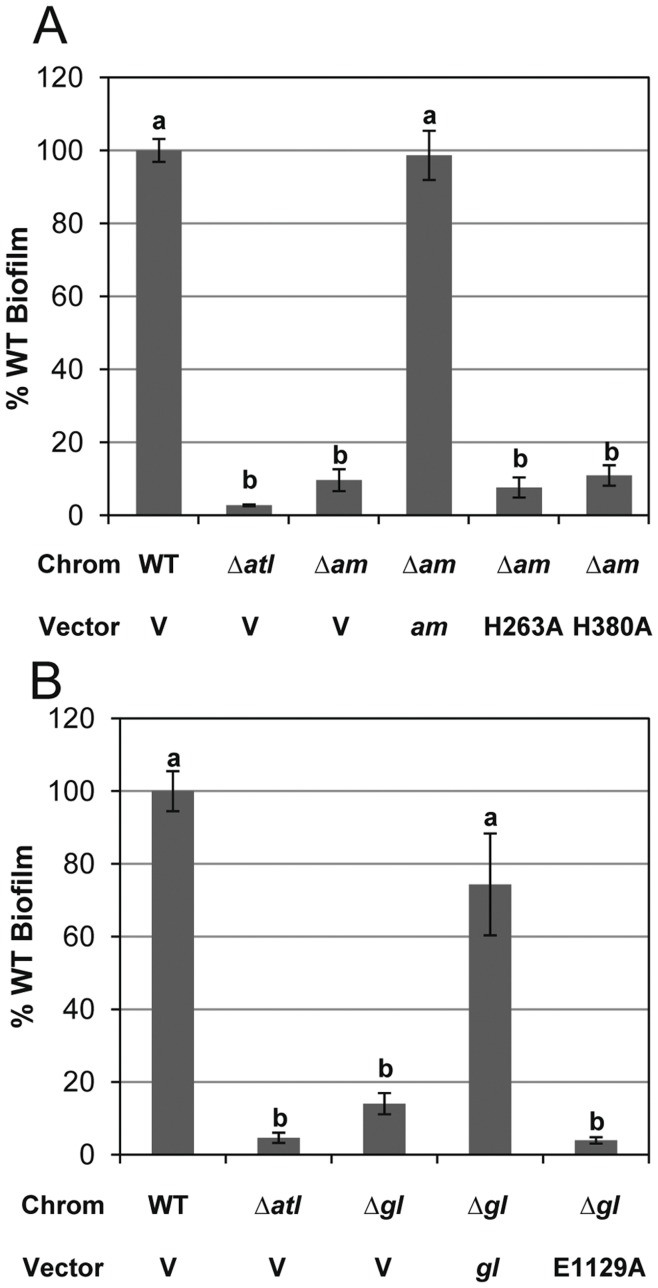
Static biofilm assays of *atl* point mutations. Quantified crystal violet stained 24-hr adherent biofilms grown in TSB supplemented with 0.25% glucose. “Chrom” denotes chromosomal genotype for UAMS-1 (WT), KB5000 (Δ*atl*), KB5002 (Δ*am*), or KB5001 (Δ*gl*). “Vector” indicates whether the strain carried vector control pJB128 (V), *atl_AM_* complement plasmid pJB111 (*am*), indicated *am* point mutation plasmids pJB122 (H263A) or pJB123 (H380A), *atl_GL_* complement plasmid pJB135(*gl*), or *gl* point mutation plasmid pJB142 (E1129A). Values were calculated using the average of the WT biofilms and represent the mean (n = 4) with standard error. Statistical analysis for both panels are denoted with lower case letters with “a” significantly different than “b” (p<0.001).

To address the function of AM and GL activity in biofilm formation, we examined the point mutant expressing strains for biofilm adherence. If these proteins serve solely as adhesins, and the region of the proteins important in adherence is independent of the enzymatic active site [6], the mutations affecting enzymatic activity should not alter biofilm formation. However, if the major function of AtlA during biofilm formation is to release matrix components such as genomic DNA, then we would expect these mutants to be unable to form an adherent biofilm. As seen in Figure 7A, while AM expressed from pJB111 was able to complement biofilm formation in the Δ*atl_AM_* mutant (producing 98.6% biomass relative to wildtype), neither AM^H263A^ (7.6% biomass; p<0.001) nor AM^H380A^ (10.9% biomass; p<0.001) could restore biofilm formation. Furthermore, while expression of GL from pJB135 partially restored the biofilm defect of the Δ*atl_GL_* mutant, the GL^E1129A^ mutation was unable to complement biofilm formation (p<0.001) (Fig. 7B). Together, these data indicate that biofilm formation in *S*. *aureus* requires both AM and GL to be active to form an adherent biofilm and support the hypothesis that the Atl-mediated release of matrix components is important in biofilm formation.

## Discussion

Unlike most murein hydrolases, the Atl murein hydrolases of *S. aureus* and *S. epidermidis* are bifunctional enzymes that possess both amidase (AM) and glucosaminidase (GL) activities. Although several studies have focused on the function of Atl [1], [2], [3], [15], none have addressed the individual contributions of the AM and GL domains in *S*. *aureus*. Thus, to gain insight into the functions of AM and GL, we generated isogenic, in-frame chromosomally-encoded AM and GL deletion mutants and tested them in assays of cell growth, lysis, and biofilm formation. Although there was no impact of the AM and GL mutations on growth rate, both mutations had a discernible effect on daughter cell separation, with the AM mutation having a greater effect than the mutation of GL. Previous studies have demonstrated that *atl* mutants grow similar to their parent strains but can be seen as larger clusters of cells when viewed microscopically [2], [17]. It has been suggested that when Atl is absent, other murein hydrolases are able to at least partially compensate for the loss of Atl activity. Possible compensating murein hydrolases include Sle1 [21], the recently described LytN [22], LytM [23], as well as greater than fifteen other proteins with putative amidase or glucosaminidase activity [22], [24].

Our results also indicate that the component of AtlA primarily responsible for the cluster phenotype is AM. Interestingly, the Δ*atl* and Δ*atl_AM_* mutants (but not the Δ*atl_GL_* mutant) produced two distinct cell cluster sizes (Fig. 2B), suggesting the presence of two populations of cells, one in which cell separation is apparently dependent on AM and another that is not. This is likely the result of activity of other *S. aureus* murein hydrolases, and suggests heterogeneity in the expression of these enzymes in the absence of AtlA. In addition, AM and GL also contributed differently to lysis with the Δ*atl_AM_* mutant exhibiting a more pronounced defect in triton X-100-induced autolysis compared to the Δ*atl_GL_* mutant. While the sensitivity of *S. aureus* peptidoglycan to AM is commonly observed by zymography [1], [2], [4], [14], [19], this is the first indication that AM might be more active than GL on live cells. Currently it is unknown why *S*. *aureus* cell walls have a higher sensitivity to AM when compared to GL. Similarly, we do not know why GL provided on a plasmid was unable to complement the autolysis defect seen in the GL mutant, despite the fact that the GL defects observed in other assays were complementable (Figs. 2A, S3C, 3, 5, 6, and 7). The possibility that the plasmid-based expression of GL is specifically inhibited by triton X-100 treatment is one explanation for this observation that is currently under consideration.

Given the role of cell lysis during biofilm development, we also examined the effects of the *atl* mutations on biofilm formation. Similar to the experiments described above, both deletion mutations exhibited phenotypes similar to the complete Δ*atl* mutant (Fig. 5), suggesting that AM and GL have non-redundant functions in biofilm formation. In agreement with this is the partial restoration of biofilm formation when expressing AM or GL in the Δ*atl* mutant. Based on the results presented here, as well as those presented elsewhere [2], [4], [5], [13], two hypotheses have been proposed for the function of Atl in biofilm formation. The first suggests that Atl serves as an adhesin, allowing cells to bind to surfaces and is based on several studies examining the ability of Atl to bind host plasma proteins. Indeed, static biofilm assays of *S*. *aureus* commonly call for pretreatment of plastic plates with dilute human plasma. Heilmann et al. [4] demonstrated that *S*. *epidermidis* AtlE exhibited strong binding to vitronectin but little interaction with fibronectin. In addition, purified truncations of *S*. *aureus* AtlA containing AM have been shown to bind to fibrinogen, fibronectin, and vitronectin [6]. An alternate hypothesis for the function of Atl in biofilm development has emerged recently and is based on the demonstration that extracellular DNA is an important matrix molecule [5], [7], [13]. In this model, Atl aids in the lysis of a subpopulation of cells, leading to genomic DNA release and biofilm formation. Several important observations support the lysis-based hypothesis. First, treatment of developing biofilms with the lysis-inhibitor, polyanethole sulfonate (PAS), prevents biofilm formation, presumably by blocking the release of genomic DNA [5], [25]. Second, disruption of the *S. aureus atlA* and *S. epidermidis atlE* results in dramatically reduced lysis (Fig. 2 and [5], [13], [14]) and the release of genomic DNA. We cannot rule out an effect of *atl* mutant-associated clustering on biofilm formation, but based on the sensitivity of *S. aureus* biofilm to nuclease, it is clear that the genomic DNA released as a result of Atl-mediated lysis is important for biofilm integrity [7], [25]. Third, point mutations inactivating the active sites of AM and GL result in the loss of biofilm formation (Fig. 7 and [5]). Importantly, the point mutations in AM are predicted to have little impact on the structure of the protein [20]. Indeed, the H263A mutation in AtlE results in loss of enzymatic activity without affecting the ability of the protein to bind a synthetic peptidoglycan substrate [20]. Fourth, it is the repeat domains and not the active site affected by the point mutations that is likely to bind host proteins [6]. Finally, purified GL has been previously shown not to bind to fibrinogen, fibronectin, or vitronectin [6] despite its clear role, as demonstrated here, in biofilm formation. Thus, the role of this murein hydrolase in biofilm formation is likely to be independent of any function as an adhesin. Together, these data demonstrate that lysis mediated by both the AM and GL domains of AtlA makes an important contribution to biofilm formation.

## Supporting Information

Figure S1 **Visualization of mutant clusters.** Wild-type UAMS-1, Δ*atl* mutant KB5000, Δ*atl_GLU_* mutant KB5001, and Δ*atl_AM_* mutant KB5002 all with pJB128 were grown to mid-exponential phase (3 hrs) in TSB with a 1∶10 media to volume ratio at 37°C with shaking (250 rpm). A sample was removed and Syto-9 added to a final concentration 5 µM of to aid in visualization. Cells were imaged by using a Zeiss 710 Confocal Laser Scanning Microscope with excitation at 488 nm and analyzed using Zen 2011 software.
(TIF)

Figure S2 **Characterization of LAC-13C and** Δ***atl*** **mutant KB4051.** A) Growth of LAC-13C (solid symbols) and Δ*atl* mutant KB4051 (empty symbols) in TSB at 1∶10 media to flask volume ratio. B) Triton X-100 induced autolysis for 3 hr cultures of LAC-13C (solid symbols) and Δ*atl* mutant KB4051 (empty symbols) grown in TSB with 1 M glucose at a 1∶10 media to flask volume ratio. C) Zymographic analysis using *Micrococcus* (Micro) or *S. aureus* (Staph) as substrates of 3 µg extracellular proteins from 3 hr cultures grown in TSB with a 1∶10 media to flask volume ratio. D) Qualitative and quantitative static biofilm following crystal violet staining. For all quantified values, data represent the mean (n = 3) with standard error.
(TIF)

Figure S3 **Cluster size of the** ***atl*** **mutants with complementation plasmids.** Wild-type UAMS-1 (black) compared to A) Δ*atl* mutant KB5000 with pJB128 (red) and KB5000 with the *atl* complement plasmid pJB141 (orange), B) Δ*atl_AM_* mutant KB5002 carrying pJB128 (green) or the *atl_AM_* complement plasmid pJB111 (brown), and C) Δ*atl_GLU_* mutant KB5001 with pJB128 (blue) or the *atl_GL_* complement plasmid pJB135 (purple) grown to mid-exponential phase (3 hrs) in TSB with a 1∶10 media to volume ratio at 37°C with shaking (250 rpm).
(TIF)

Figure S4 **Zymographic and western blot analyses of the** ***atl*** **point mutants.** Three µg of total extracellular proteins from the *atl_AM_* mutant (KB5002) and the *atl_GL_* mutant (KB5001) with wild-type and point mutation allele expressing plasmids were examined in zymography gels containing either *S*. *aureus* (upper left panel) or *Micrococcus* (upper right panel) cells as a substrate. Proteins were also analyzed in western blot experiments using either anti-amidase (left) or anti-glucosaminidase (right) antibodies as probes. “Chrom” denotes the chromosomal genotype for KB5002 (Δ*am*), or KB5001 (Δ*gl*). “Vector” indicates whether the strain carried the *atl_am_* complement plasmid, pJB111 (*am*), the indicated *am* point mutation plasmids, pJB122 (H263A) or pJB123 (H380A), the *atl_gl_* complement plasmid, pJB135(*gl*), or the *gl* point mutation plasmid, pJB142 (E1129A).
(TIF)

Table S1 **Select bacterial strains and plasmids used in this study.**
(DOCX)

Table S2 **Plasmid construction and oligonucleotides.**
(DOCX)
