# Supplementary figures and images for: Contribution of the Staphylococcus aureus Atl AM and GL Murein Hydrolase Activities in Cell Division, Autolysis, and Biofilm Formation
Source: PLoS One. 2012 Jul 31;7(7):e42244. doi: 10.1371/journal.pone.0042244 (PMC3409170; doi:10.1371/journal.pone.0042244)

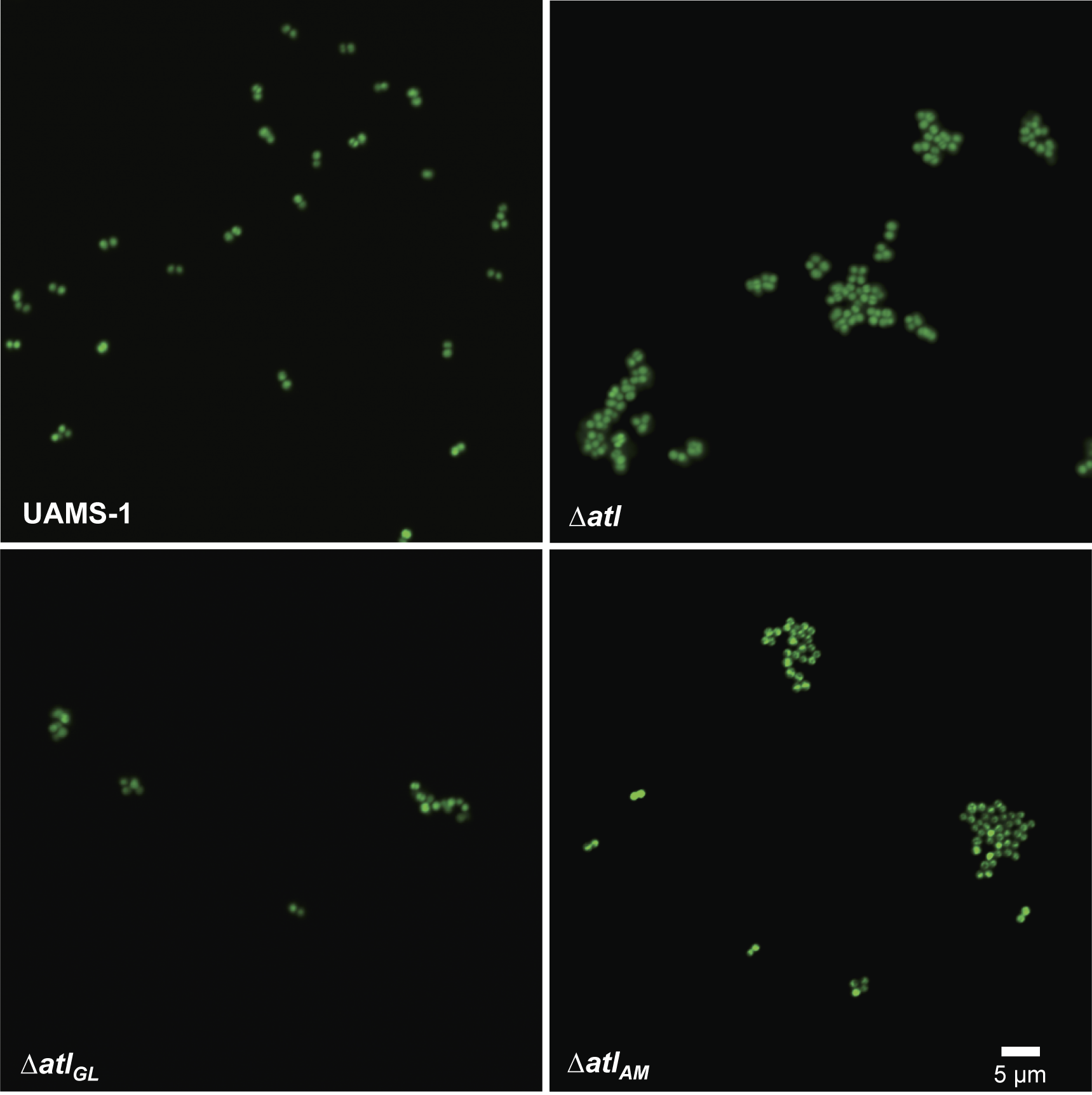

Supplement: Figure S1 — Visualization of mutant clusters. Wild-type UAMS-1, Δatl mutant KB5000, ΔatlGLU mutant KB5001, and ΔatlAM mutant KB5002 all with pJB128 were grown to mid-exponential phase (3 hrs) in TSB with a 1∶10 media to volume ratio at 37°C with shaking (250 rpm). A sample was removed and Syto-9 added to a final concentration 5 µM of to aid in visualization. Cells were imaged by using a Zeiss 710 Confocal Laser Scanning Microscope with excitation at 488 nm and analyzed using Zen 2011 software. (TIF) [file pone.0042244.s001.tif]

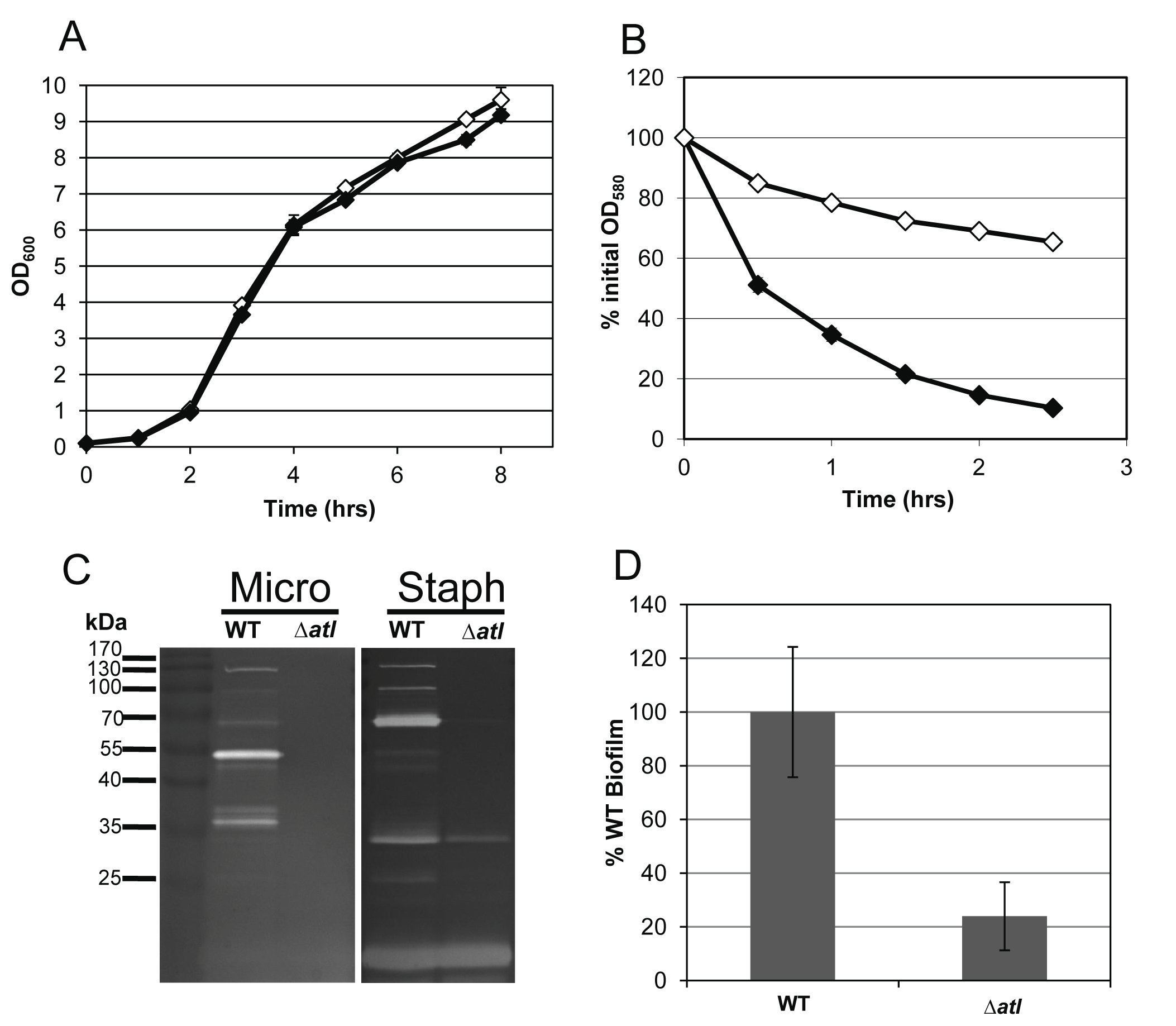

Supplement: Figure S2 — Characterization of LAC-13C and Δatl mutant KB4051. A) Growth of LAC-13C (solid symbols) and Δatl mutant KB4051 (empty symbols) in TSB at 1∶10 media to flask volume ratio. B) Triton X-100 induced autolysis for 3 hr cultures of LAC-13C (solid symbols) and Δatl mutant KB4051 (empty symbols) grown in TSB with 1 M glucose at a 1∶10 media to flask volume ratio. C) Zymographic analysis using Micrococcus (Micro) or S. aureus (Staph) as substrates of 3 µg extracellular proteins from 3 hr cultures grown in TSB with a 1∶10 media to flask volume ratio. D) Qualitative and quantitative static biofilm following crystal violet staining. For all quantified values, data represent the mean (n = 3) with standard error. (TIF) [file pone.0042244.s002.tif]

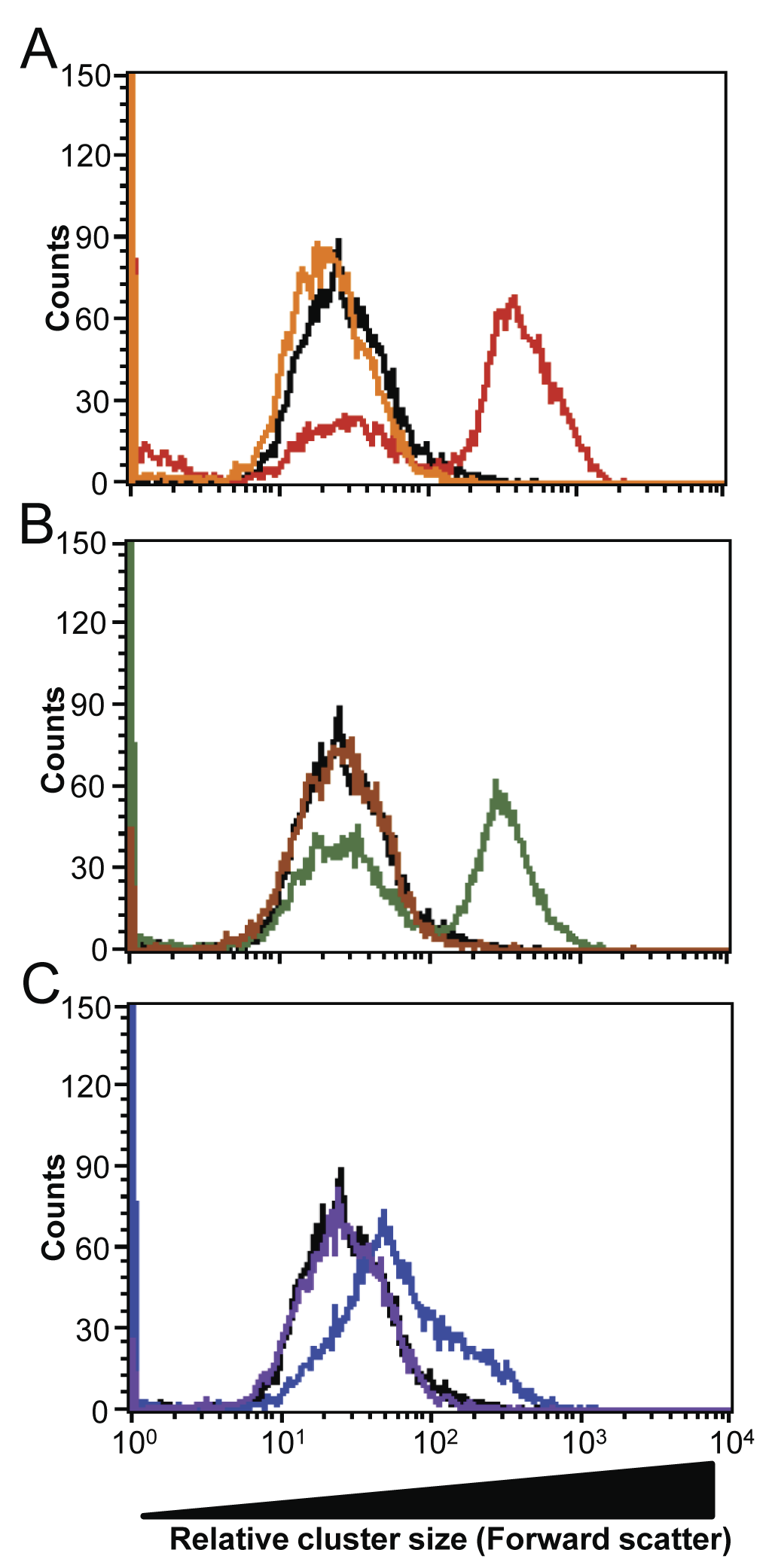

Supplement: Figure S3 — Cluster size of the atl mutants with complementation plasmids. Wild-type UAMS-1 (black) compared to A) Δatl mutant KB5000 with pJB128 (red) and KB5000 with the atl complement plasmid pJB141 (orange), B) ΔatlAM mutant KB5002 carrying pJB128 (green) or the atlAM complement plasmid pJB111 (brown), and C) ΔatlGLU mutant KB5001 with pJB128 (blue) or the atlGL complement plasmid pJB135 (purple) grown to mid-exponential phase (3 hrs) in TSB with a 1∶10 media to volume ratio at 37°C with shaking (250 rpm). (TIF) [file pone.0042244.s003.tif]

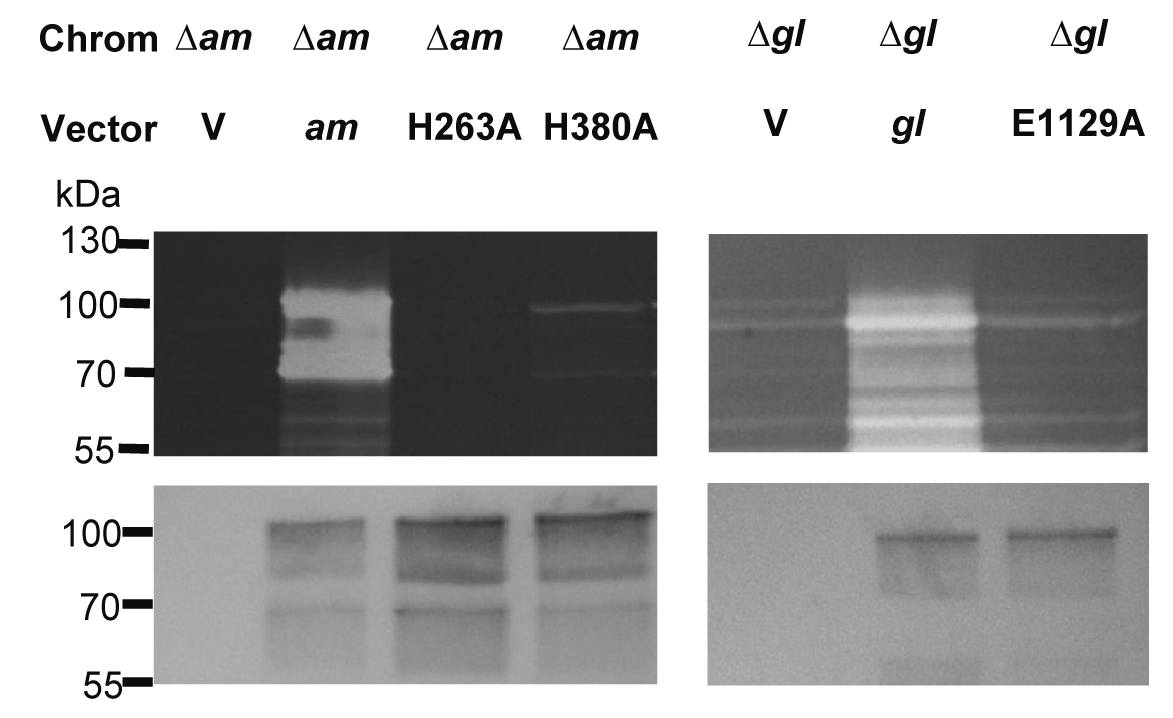

Supplement: Figure S4 — Zymographic and western blot analyses of the atl point mutants. Three µg of total extracellular proteins from the atlAM mutant (KB5002) and the atlGL mutant (KB5001) with wild-type and point mutation allele expressing plasmids were examined in zymography gels containing either S. aureus (upper left panel) or Micrococcus (upper right panel) cells as a substrate. Proteins were also analyzed in western blot experiments using either anti-amidase (left) or anti-glucosaminidase (right) antibodies as probes. “Chrom” denotes the chromosomal genotype for KB5002 (Δam), or KB5001 (Δgl). “Vector” indicates whether the strain carried the atlam complement plasmid, pJB111 (am), the indicated am point mutation plasmids, pJB122 (H263A) or pJB123 (H380A), the atlgl complement plasmid, pJB135(gl), or the gl point mutation plasmid, pJB142 (E1129A). (TIF) [file pone.0042244.s004.tif]
