# Supplementary material for: Contribution of the Staphylococcus aureus Atl AM and GL Murein Hydrolase Activities in Cell Division, Autolysis, and Biofilm Formation
Source: PLoS One. 2012 Jul 31;7(7):e42244. doi: 10.1371/journal.pone.0042244 (PMC3409170; doi:10.1371/journal.pone.0042244)
Supplement: Table S1 — Select bacterial strains and plasmids used in this study. (DOCX) [file pone.0042244.s005.docx]

Table S1. Select bacterial strains and plasmids used in this study

| Strain or plasmid | Relevant characteristics | Source or reference |
| --- | --- | --- |
| **Bacterial strains** |  |  |
| ***S. aureus*** |  |  |
| LAC | Wildtype MRSA abscess isolate | [[1](#_ENREF_1)] |
| LAC-13C | LAC cured of LAC-p03 | This study |
| RN4220 | Highly transformable restriction-deficient strain | [[2](#_ENREF_2)] |
| UAMS-1 | Wildtype osteomylitis isolate | [[3](#_ENREF_3)] |
| KB4051 | LAC-13C Δ*atlA* | This study |
| KB5000 | UAMS-1 Δ*atlA* | This study |
| KB5001 | UAMS-1 Δ*atlA_GL_* | This study |
| KB5002 | UAMS-1 Δ*atlA_AM_* | This study |
|  |  |  |
|  |  |  |
| **Plasmids** |  |  |
| pJB89 | UAMS-1 Δ*atlA*  allelic exchange plasmid | This study |
| pJB96 | UAMS-1 Δ*atlA_GL_*  allelic exchange plasmid | This study |
| pJB100 | UAMS-1 Δ*atlA_AM_*  allelic exchange plasmid | This study |
| pJB101 | LAC-13C Δ*atlA*  allelic exchange plasmid | This study |
| pJB111 | UAMS-1 *atlA_AM_*  complement plasmid | This study |
| pJB122 | UAMS-1 *atlA_AMH263A_*  complement plasmid | This study |
| pJB123 | UAMS-1 *atlA_AMH380A_*  complement plasmid | This study |
| pJB128 | Insertless complement plasmid control | This study |
| pJB135 | UAMS-1 *atlA_GL_*  complement plasmid | This study |
| pJB141 | UAMS-1 *atl*  complement plasmid | This study |
| pJB142 | UAMS-1 *atl_GLE1129A_*  complement plasmid | This study |

**References**

1. Voyich JM, Braughton KR, Sturdevant DE, Whitney AR, Said-Salim B, et al. (2005) Insights into mechanisms used by *Staphylococcus aureus* to avoid destruction by human neutrophils. Journal of immunology 175: 3907-3919.

2. Kreiswirth BN, Lofdahl S, Betley MJ, O'Reilly M, Schlievert PM, et al. (1983) The toxic shock syndrome exotoxin structural gene is not detectably transmitted by a prophage. Nature 305: 709-712.

3. Gillaspy AF, Hickmon SG, Skinner RA, Thomas JR, Nelson CL, et al. (1995) Role of the accessory gene regulator (agr) in pathogenesis of staphylococcal osteomyelitis. Infection and Immunity 63: 3373-3380.
