# Supplementary material for: Contribution of the Staphylococcus aureus Atl AM and GL Murein Hydrolase Activities in Cell Division, Autolysis, and Biofilm Formation
Source: PLoS One. 2012 Jul 31;7(7):e42244. doi: 10.1371/journal.pone.0042244 (PMC3409170; doi:10.1371/journal.pone.0042244)
Supplement: Table S2 — Plasmid construction and oligonucleotides. (DOCX) [file pone.0042244.s006.docx]

Table S2. Plasmid construction and oligonucleotides

| Plasmid or oligonucleotide | Relevant characteristics^a^ | Source or reference |
| --- | --- | --- |
| **Plasmid** |  |  |
| pCL10 | Temperature-sensitive cloning vector, *ampR*, *chmR* | [[1](#_ENREF_1)] |
| pCL52.2 | Temperature-sensitive cloning vector, *specR*, *tetR* | [[1](#_ENREF_1)] |
| pCN51 | pT181*cop*-*wt repC ermR ampR* ColE1 *oriV blaZTT* | [[2](#_ENREF_2)] |
| pCR-Blunt | PCR cloning vector; ColE1 *oriV*, *kanR* | Invitrogen |
| pJB28 | PvuII/EcoRI-digested PCR product containing R6Kγ *oriV* (primers JBR6KORI1 and JBR6KORI2, pVSV208 template) in AfeI/PvuII-digested pCL52.2 | This study |
| pJB44 | pJB28 NheI/AvrII fragment containing R6Kγ *oriV* and SpecR self-ligated | This study |
| pJB89 | EcoRI/BamHI-digested PCR product containing *atl* upstream (primers JBATL10 and JBATL11, UAMS-1 template) and BamHI/SalI-digested PCR product containing *atl* downstream (primers JBATL12 and JBATL13, UAMS-1 template) in EcoRI/SalI-digested pCL52.2 | This study |
| pJB96 | EcoRI/BamHI-digested PCR product containing partial *atl* (primers JBATL18 and JBATL19, UAMS-1 template) in same sites of pJB89 | This study |
| pJB98 | BamHI/SalI-digested PCR product containing downstream of *atl_AM_* (primers JBATL22 and JBATL23, UAMS-1 template) in same sites of pCL52.2 | This study |
| pJB99 | EcoRI/BamHI-digested PCR product containing *atl* upstream (primers JBATL10L and JBATL11, LAC-13C template) and BamHI/SalI-digested PCR product containing *atl* downstream (primers JBATL12 and JBATL13, LAC-13C template) in EcoRI/SalI-digested pCL10 | This study |
| pJB100 | EcoRI/BamHI-digested PCR product containing upstream of *atl_AM_* (primers JBATL24 and JBATL25, UAMS-1 template) in same sites of pJB98 | This study |
| pJB101 | BamHI-digested pJB99 self-ligated | This study |
| pJB103 | AvrII/XhoI-digested pCN51 fragment containing all but ermR, Klenow-treated, and self-ligated | This study |
| pJB104 | AvrII/XhoI-digested pCN51 fragment containing ermR, Klenow-treated, in Klenow-treated, NheI/BglII-digested fragment containing R6Kγ oriV and SpecR of pJB44 | This study |
| pJB106 | PstI-fragment of PCR product (primers JBATL14 and JBATL16, UAMS-1 template) containing partial *atl* and upstream in SmaI/PstI-digested pJB104 | This study |
| pJB107 | PstI-fragment of PCR product (primers JBATL14 and JBATL16, UAMS-1 template) containing partial *atl_AM_* in SmaI/PstI-digested pJB103 | This study |
| pJB110 | PCR product containing partial *atl_AM_* (Primers JBATL26 and JBATL27, UAMS-1 template) in pCR-Blunt | This study |
| pJB111 | *atlA_AM_* complementation plasmid; PstI-digested pJB106 and pJB107 ligated together | This study |
| pJB113 | Self-ligated PCR product (Primers JBATL30 and JBATL31, pJB110 template) | This study |
| pJB114 | Self-ligated PCR product (Primers JBATL32 and JBATL33, pJB110 template) | This study |
| pJB120 | pJB113 NcoI/PstI fragment containing *atl_AMH263A_* same sites of pJB107 | This study |
| pJB121 | pJB114 NcoI/PstI fragment containing *atl_AMH380A_* same sites of pJB107 | This study |
| pJB122 | PstI-digested pJB106 and pJB120 ligated together | This study |
| pJB123 | PstI-digested pJB106 and pJB121 ligated together | This study |
| pJB128 | PstI-digested pJB103 and pJB104 ligated together | This study |
| pJB129 | PCR product (primers JBATL2 and JBATL3, KB5002 template) containing *atl_GL_* in pCR-Blunt | This study |
| pJB133 | EcoRI/PstI fragment containing *atl_GL_* from pJB129 in same sites of pJB103 | This study |
| pJB135 | *atlA_GL_* complementation plasmid; PstI-digested pJB106 and pJB133 ligated together | This study |
| pJB138 | PstI/BamHI fragment PCR product containing partial *atl* (Primers JBATL14 and JBATL15, UAMS-1 template) in same sites of pJB103 | This study |
| pJB139 | Self-ligated PCR product (Primers JBATL35 and JBATL36, pJB129 template) | This study |
| pJB140 | pJB139 EcoRI/PstI fragment containing *atl_GLE1129A_* in same sites of pJB103 | This study |
| pJB141 | *atlA* complementation plasmid; PstI-digested pJB106 and pJB138 ligated together | This study |
| pJB142 | PstI-digested pJB106 and pJB140 ligated together | This study |
| pVSV208 | Source of R6Kγ oriV | [[3](#_ENREF_3)] |
|  |  |  |

| **Oligonucleotides^b^** |  |  |
| --- | --- | --- |
| JBATL1^c^ | GGTTAAATACGCACAGGTGTATAAAACAGGTAC | This study |
| JBATL2^c^ | GGGATGACTTTTGATCCTATGTTCATGTTGC | This study |
| JBATL3^c^ | CACCTGTTGCACCTAAAGCTAAAACTGAAGC | This study |
| JBATL4^c^ | GGAAATGGTACAGTATGGACTCACTACGC | This study |
| JBATL5^c^ | CTGGTTCTGGAAACCAAACATTTAAGGCTTC | This study |
| JBATL6^c^ | GTAAAGCATATTTAGCTGTACCTGCTGCACC | This study |
| JBATL7^c^ | GGTTATTACTATGTAACACCAAATTCTGATACAGC | This study |
| JBATL8^c^ | CTTTACTTACCCAACCAGATTTACCATTCACAGAGC | This study |
| JBATL9^c^ | TGTATCATGAACAACGATACCTTCAGGACGAC | This study |
| JBATL10 | *ccgaattc*gtcgcagcagctttagaagtgacagatg | This study |
| JBATL10L | *ccgaattc*GTTGCAGCAGCTTTAGAAGTAACTGATG | This study |
| JBATL11 | *CGGATCC*CGCCATTCTATTTATTACTCCTAACATTTATT | This study |
| JBATL12 | *cggatcc*taagcaacatgaacataggatcaaaagtcatc | This study |
| JBATL13 | *GCGTCGAC*TCGCCACTAGTGTAGGGTTGTAACATATGTC | This study |
| JBATL14 | *CCGAGCT*CTTGAGGAAGGCATCGAGCATATTGAAATGAC | This study |
| JBATL15 | *CCGGATCC*GAATCATGAATTACAAGCAAAAGTAGCGGTG | This study |
| JBATL16^c^ | *GGCTCGAGT*TAAGCTTTTACAGCAGTTTTTGGTTGTGC | This study |
| JBATL17^c^ | *CCCTCGA*GCAACATGAACATAGGATCAAAAGTCATCC | This study |
| JBATL18 | *CCGAATTC*GCGATGTGGTTTACAACACAGCTAAATCAC | This study |
| JBATL19 | *CCGGATCC*AGCTTTTACAGCAGTTTTTGGTTGTGC | This study |
| JBATL20^c^ | *CCGAATTC*GGATATGATAATGGACAGCCAGTTGCCAC | This study |
| JBATL21^c^ | *CCGGATCC*CGTTTTAGGTGCTGTTGTGTTTGTTGACTTTG | This study |
| JBATL22 | *CCGGATCC*GCTGCACCTAAAAAAGCTGTAGCACAAC | This study |
| JBATL23 | *CCGTCGAC*CTTGAGCTAAACGCTTCGTATCCATTGC | This study |
| JBATL24 | *CCGGATCC*ACTCGTTTTAGGTGCTGCAGCGGCTGATCTTG | This study |
| JBATL25 | *CCGAATTC*CAAACTACCATCAATGGTTGCATTAACGC | This study |
| JBATL26 | ccgctgcagcacctaaaacgagtttgc | This study |
| JBATL27 | GCGTACCCCATGGCGCCACTTTAC | This study |
| JBATL28 | *CCCTGC*AGTGGCGCCATGGGGTACGCAATCTACAAC | This study |
| JBATL29 | *GGAAGC*TTAGCATTAATTTGAGCAACACCGTTTAATGATG | This study |
| JBATL30 | P-AACAACGATACCTTCAGGACGACCTACAC | This study |
| JBATL31 | P-*GCA*GATACAGCTAATGATCGTTCGACGATAAATG | This study |
| JBATL32 | P-GTCCGTACCACCTAAATATTTACTTACAGCGTAGTG | This study |
| JBATL33 | P-*GCA*GCCGATCCACATGGATATTTAAGAAGTC | This study |
| JBATL34^c^ | cgctgcagcacctaaaacgagtggatccgctgcac | This study |
| JBATL35 | P-TAATAGGGCATGTGAGATAAGATAAACTTCATTAATGC | This study |
| JBATL36 | P-*gCa*acaggtaacggtacttctcaattagcaaaaggtgc | This study |
| JBR6KORI1 | *ccg*aattcccatgtcagccgttaagtgttcc | This study |
| JBR6KORI2 | *cccagct*GGCCACGATGCGTCCGGCGTAGAGGATC | This study |
|  |  |  |

^a^ Antibiotic resistance abbreviations used: *ampR*, ampicillin resistance (*bla*); *chmR*, chloramphenicol resistance; *ermR*, erythromycin resistance; *kanR*, kanamycin resistance (*aph*); *tetR*, tetracycline resistance (*tetM*), and *specR*, spectinomycin resistance.

^b^ Oligonucleotides sequences are provided in the 5’-3’ orientation. "P-" indicates 5' phosphorylation. Italics indicate non-homologous sequences added for cloning purposes.

^c^ Oligonucleotide used for sequencing only.

**References**

1. Sau S, Sun J, Lee CY (1997) Molecular characterization and transcriptional analysis of type 8 capsule genes in *Staphylococcus aureus*. Journal of Bacteriology 179: 1614-1621.

2. Charpentier E, Anton AI, Barry P, Alfonso B, Fang Y, et al. (2004) Novel cassette-based shuttle vector system for gram-positive bacteria. Applied and Environmental Microbiology 70: 6076-6085.

3. Dunn AK, Millikan DS, Adin DM, Bose JL, Stabb EV (2006) New rfp- and pES213-derived tools for analyzing symbiotic *Vibrio fischeri* reveal patterns of infection and lux expression in situ. Appl Environ Microbiol 72: 802-810.
